# World Hepatitis Day — July 28, 2014

**Published:** 2014-07-25

July 28, 2014, marks the 4th annual World Hepatitis Day. Nearly 400 million persons are living with hepatitis B or hepatitis C, and more than 1 million die annually as a result of their infection. This year, the 67th World Health Assembly (WHA) reaffirmed the global commitment to prevent and control viral hepatitis through the passage of resolution WHA 67.6 (1), which calls for raising public awareness, improving surveillance, strengthening prevention interventions, and increasing access to care and treatment services.

Blood transfusions save lives, and globally more than 100 million units of blood are donated annually. Ensuring access to safe blood is a key strategy for the prevention of hepatitis B and C. In many of the poorest countries of the world, less than 50% of the blood supply comes from voluntary, unpaid donors that were adequately screened for transfusion transmitted infections, including hepatitis B and C.

Prevention and control of hepatitis remains a major challenge in sub-Saharan Africa. This issue of *MMWR* includes a report from sub-Saharan Africa describing substantial increases in the number of blood units donated and screened for hepatitis B and C during the last decade. Despite these gains, the report demonstrates that the risk for transmission of hepatitis B and C through transfusion persists in many countries in the region. It is estimated that in sub-Saharan Africa, more than 45,000 hepatitis B virus or hepatitis C virus infections are transmitted through contaminated transfusions annually (2).

Resources and information about World Hepatitis Day are available at http://www.cdc.gov/hepatitis/worldhepday.htm.
